# Weight Shift Movements of a Social Mediator Robot Make It Being Recognized as Serious and Suppress Anger, Revenge and Avoidance Motivation of the User

**DOI:** 10.3389/frobt.2022.790209

**Published:** 2022-02-28

**Authors:** Yohei Noguchi, Hiroko Kamide, Fumihide Tanaka

**Affiliations:** ^1^ Department of Intelligent Interaction Technologies, University of Tsukuba, Ibaraki, Japan; ^2^ Institute of Innovation for Future Society, Nagoya University, Aichi, Japan; ^3^ Faculty of Engineering, Information and Systems, University of Tsukuba, Ibaraki, Japan

**Keywords:** social mediator robot, weight shift, handheld device, text messaging, anger suppression, inducing forgiveness, seriousness

## Abstract

Humans can become aggressive during text messaging. To maintain a healthy interpersonal relationship through text messaging, our negative mental states, such as anger, have to be well-controlled. This paper discusses the use of a handheld social robot deployed as a mediator in text messaging between humans. The robot is equipped with a movable weight inside its body. By controlling the movement of the internal weight during the time when the robot speaks out messages received from a human sender, we hypothesize that the psychological state of a receiver who holds the robot can be affected (for example, he/she will listen to the messages more seriously). In a controlled study (*n* = 94), in which participants were manipulated to be frustrated by using a context scenario, we studied the effect of three dialogue scripts with/without weight shifts. Results showed that introducing weight shifts together with the robot speech suppressed on average 23% of the user’s anger. However, only 3.5% of the anger was suppressed when the weight shifts were not applied. Additionally, in cases where the robot showed empathy to the user in words with weight shifts, the user’s revenge urge was successfully reduced by 22%. There was almost no effect confirmed when the weight shifts were not applied. A similar effect was also found in avoidance motivation: 15% of the avoidance motivation was reduced if weight shifts were applied. The reductions in revenge and avoidance motivation are considered important factors for human forgiveness. Therefore, our findings provide experimental evidence that weight shifts can be an effective expression modality for mediator robots, from the perspective of not only suppressing the user’s anger but also by inducing forgiveness during messaging.

## 1 Introduction

Text messaging devices (such as mobile phones) brought huge benefits to interpersonal communication in the last three decades. The advent of these devices has made people’s remote communication become instant. Furthermore, compared with other classic devices based on typing, modern devices with voice recognition capabilities provide a more efficient message creation process to users (such as smart speakers). Also, social robots have been employed as text messaging devices. Research evidences show that social robots that serve as mediators between humans encourage remote communication of the users (Kobayashi et al., 2017; Sasama et al., 2011; Noguchi et al., 2020). Moreover, such a mediator robot can influence the message content. For instance, Noguchi et al. (2020) reported that the depth of self-disclosure of the elderly to their remote family members was significantly affected by the behaviors of a mediator robot.

Studies on computer-mediated communication (CMC) revealed that the users’ hostile, aggressive, and uninhibited communicative behaviors, called flaming, were more frequently observed in text messaging than face-to-face and videoconferencing (e.g., Lea et al., 1992; Kiesler et al., 1985; Castellá et al., 2000). To maintain healthy interpersonal relationships by reducing the user’s aggressive behaviors, a mediator robot should be designed so that it can suppress the user’s anger and other interpersonal motivations (e.g., revenge and avoidance motivations). The reduction in both revenge and avoidance motivations is considered the central concept for inducing human forgiveness (McCullough et al., 1998). However, from this viewpoint, the introduction of mediator robot has never been explored.

**FIGURE 1:**
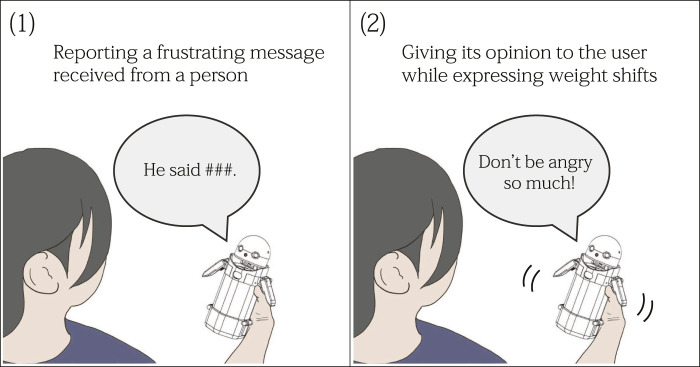
Robot intervention. A mediator robot reports a frustrating message received from a person followed by giving its opinion. The robot can also express internal weight movements (weight shifts). By expressing appropriate weight shifts along with its speech, we hypothesize that the serious intention of the robot can be enhanced, thereby influencing the user’s negative affective state.

This study investigates the behavior of a mediator robot that is particularly effective for users receiving frustrating messages from other people. We assume a human-robot interaction (HRI) scenario in which a robot verbally gives its opinion to such users while expressing non-verbal signals (in this paper, we call these series of behaviors “robot intervention”) (Figure 1). As the non-verbal expression modality of robot intervention, we focused on a haptic-based interaction channel using internal weight movements (weight shifts). To this end, we introduce a handheld robot, called OMOY, equipped with a movable weight actuated by mechanical components inside its body. Through the weight shifts, the robot expresses its emotions and intentions to the user holding it (Noguchi and Tanaka, 2020). Furthermore, using weight shifts to communicate may influence the users’ social judgments and decisions. Following psychology literature suggesting that the abstract concept of importance is grounded in bodily experiences of weight (Jostmann et al., 2009), recognized importance of robot’s speech contents may be enhanced when the users feel heaviness in weight shifts.

In this paper, we report an HRI study involving 94 participants. By using a context scenario, the participants were manipulated to be frustrated by a message sent from another person. OMOY reported the message while providing its opinion to each participant on how to deal with their frustrations. One of the three opinions was presented to each participant: (1) OMOY suggested that the participant should suppress their anger, (2) OMOY showed empathy to the participant, and (3) OMOY avoided providing some particular comments (the robot tried to be a bystander). During the expression of its opinion, OMOY exhibited weight shifts to half the number of participants. Then, we questioned the participants and analyzed the obtained results to answer the following research questions:

• RQ1: How much can the user’s anger, revenge and avoidance motivations be suppressed by the mediator robot exhibiting weight shifts?
• RQ2: Does exhibiting weight shifts affect the user’s recognition about the seriousness of the robot? If so, does this feature relate with the results for RQ1?
• RQ3: How does the effect of weight shifts differ depending on the opinion type?

The remainder of this paper is organized as follows: Section 2 presents related works. Section 3 explains the robot platform used in this study. Section 4 describes the study method. Section 5 reports the study results. Section 6 provides general discussions and limitations of the study, and Section 7 presents our conclusion.

## 2 Related Works

### 2.1 Social Robot as a Mediator for Humans

In the context of CMC, researchers have explored a wide range of the social effects of communication between people using network-connected digital devices to exchange messages (e.g., text messaging, social network site interactions, videoconferencing) (Thurlow et al., 2004). Among the CMC channels, text messaging has been majorly studied because of its unique features attributed to the limited non-verbal information for exchange and asynchronicity (Walther, 1996; Joinson, 2001). As important implications on the use of text messaging, researchers identified the users’ hostile, aggressive, and uninhibited communicative behaviors, called flaming, increased compared to those of face-to-face and videoconferencing (e.g., Lea et al., 1992; Kiesler et al., 1985; Castellá et al., 2000). One explanation on the presence of these behaviors has been given based on deindividuation. Kiesler et al. (1984) discussed the phenomenon of the deindividuation in CMC as a situation where “social standards will be less important and communication will be more impersonal and more free because the rapid exchange of text, the lack of social feedback, and the absence of norms governing the social interaction redirect attention away from others and toward the message itself.” Such situations reduce users’ public self-awareness (Matheson and Zanna, 1988), and more impulsive and assertive behavior not considering the recipients’ feelings can be promoted (Castellá et al., 2000).

To overcome this issue while ensuring the benefit of using text messaging, we consider an approach that involves an intelligent supportive agent, such as a dialogue robot for text messaging. By providing interventions from the robot, such as showing empathy to the users, the quality of messages can be influenced. As an example, from a case study conducted to encourage elderly self-disclosure to their remote family members by applying a social mediator robot, the property of its expressions (i.e., personality traits and quantity of social behaviors) significantly influenced the depth of the elderly self-disclosure (Noguchi et al., 2020). The key finding of the study is that the information disclosed by users depends on the behaviors of the mediator robot locally interacting with them. Furthermore, recently, Hancock et al. (2020) conceptualized artificial intelligence-mediated communication (AI-MC) as a modern extension of CMC and envisioned it as a possible future communication style for humans: defined as “interpersonal communication in which an intelligent agent operates on behalf of a communicator by modifying, augmenting, or generating messages to accomplish communication goals.” Some of our devices have been embedded with some agents to mediate our messaging (e.g., Gmail smart reply). Therefore, if more and more robots and AI agents mediate our communication, they can be applied as a new standard in the future.

### 2.2 Weight Shift as a Novel Expression Modality for Social Robot

Expression modalities of social robots have been widely explored in human-robot interaction (HRI). Many social robots have been designed to convey their internal states by speech dialogues and non-verbal signals. For example, facial expressions (Todo, 2018), body movements (Nakata et al., 1998), sounds (Jee et al., 2009), sweats (Guo and Tanaka, 2020), colors (Terada et al., 2012), vibrations (Song and Yamada, 2017), and their combinations (Löffler et al., 2018; Song and Yamada, 2017) have been used as non-verbal expression modalities for social robots. Recently, dynamic haptic parameters, such as changes in skin temperature (Peña and Tanaka, 2020; Nie et al., 2012; Block and Kuchenbecker, 2018), texture (Hu and Hoffman, 2019), and SwarmHaptics (Kim and Follmer, 2019) have been discussed in the literature. Interactions using these haptic modalities are particularly useful in complementing the expressiveness of robots designed with few anthropomorphic features and configurations. However, besides (Noguchi and Tanaka, 2020), no study on robotics research has investigated the use of internal weight movements (weight shifts) in robot expression.

From the technical perspective of weight shifts, handheld devices with weight-shifting functions have been mainly studied in the field of virtual reality (VR). Since the relationship between the presented torque and the human perception of heaviness was found by Woodruff and Helson (1965), researchers have designed devices based on the principle. For example, TorqueBAR, Shifty, and Transcalibar are haptic feedback devices equipped with 1D or 2D translational weight-shifting mechanisms. Changing the distribution of the internal weight enhanced the users’ perception of virtual objects (Swindells et al., 2003; Zenner and Krüger, 2017; Shigeyama et al., 2019). SWISH is a human-computer interface capable of simulating dynamic fluid behavior in VR or augmented reality (Sagheb et al., 2019). It mimics the shifts of the center of gravity of a fluid vessel with a responsive motor actuation integrated with virtual fluid simulation. From other perspectives on weight shift use, haptic displays for mobile phones have been discussed. Hemmert et al. (2010) proposed Weight-Shifting Mobile, a mass-shift based system, for haptic actuation in mobile phones. They suggested that changing of the gravitational properties of the device provides beneficial applications, including augmenting digital content with physical mass, ambient displays, and haptically augmented wayfinding. Compared with previous works, studying weight shifts as an expression modality of social robots is novel. Additionally, we focus on the combining speech dialogues and discuss its effects on human perception, an unexplored area in both HRI and VR.

From the embodied perspective on cognition, the weight-based interaction channel of handheld devices can influence users’ judgements and decisions. Jostmann et al. (2009) suggested that the abstract concept of importance is grounded in bodily experiences. Some important results were obtained from the studies conducted: holding a heavy clipboard increased judgments on the monetary value and made participants consider fair decision-making procedures as more important. Similarly, Ackerman et al. (2010) explored how incidental heaviness sensation affects human decisions. As a result, heavy objects made job candidates appear more important: participants using heavy clipboards rated the candidate as displaying a more serious interest in the position. These findings suggest that, in principle, communicating through weight conveys the seriousness of the robot. Therefore, if an appropriate weight shift is expressed along with robot speech, the user may listen to the speech more seriously than the case no weight shift is expressed.

## 3 Robot Platform

**FIGURE 2:**
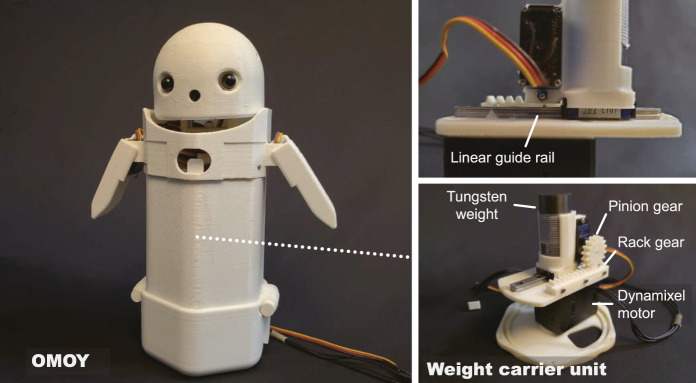
Robot platform, named OMOY, used in this study. A 250 g tungsten weight is attached to the weight carrier unit, which allows the weight to move along a 2D planar space. We modified the weight carrier unit from the original version reported in Noguchi and Tanaka (2020): a linear guide rail and a Dynamixel motor were installed.

The platform used in this study is OMOY, a handheld robot specifically developed to study weight shifts and speech dialogues. OMOY presents weight shifts by moving its internal weight. More specifically, a 250 g tungsten weight is attached to a weight carrier unit, allowing the weight to move along a 2D planar space, thus, the robot provides the weight shift perception to the user who holds the robot. For this study, we provide a little modification to this unit. A linear metal guide rail was applied to achieve smoother translational movements and a Dynamixel XC430-W150T servo motor (1.6 Nm at a 12 V with 1.4 A) was installed on the rotational mechanism to achieve more durability (Figure 2). Besides the weight shift expressions, OMOY has basic expression capabilities of hand/head gestures and speech dialogues, similar to other social robots. The dimensions of OMOY are approximately 240 mm (H) × 125/230 mm (arm closed/arm open) (W) × 95 mm (D) and it weights 725 g^1^. The appearance of the OMOY was designed to minimize the influence of prior knowledge of existing commercial robots, thus, it has a simple appearance and body shape. A full description of the mechanical design is found in (Noguchi and Tanaka, 2020).

## 4 Methods

**FIGURE 3:**
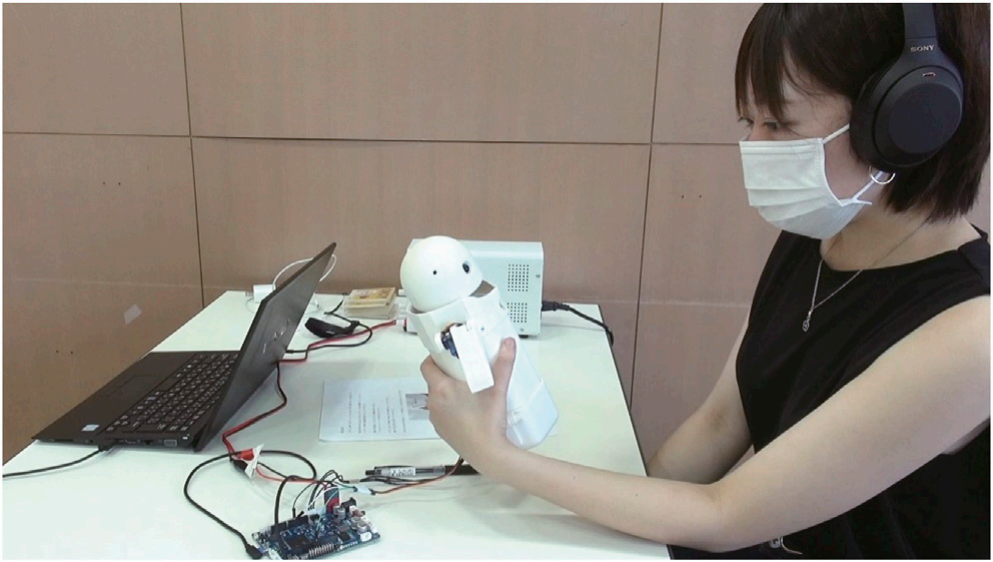
The experiment was conducted at a booth constructed in a cafeteria in the University of Tsukuba. Each participant was asked to hold OMOY and tested one of its behaviors generated by speech and weight shifts. An experimenter took a seat in front of the participant.

We investigated the effect of robot behaviors on the user’s negative affective states in a text messaging using weight shifts. Thus, we conducted an HRI study (*n* = 94) in which OMOY served as a remote communication interface between humans (Figure 3). Participants were provided a specific context scenario that got them frustrated due to a message from their friend (described in Section 4.1). The message was read out by OMOY using its voice-synthesizing function, thus, it did not contain any social cues linked to the message sender. Upon reporting the message, OMOY verbally gave its opinion randomly chosen from the three dialogue scripts: (1) the robot suggests that the participant should suppress his/her anger, (2) the robot shows its empathy to the participant, or (3) the robot avoids providing some particular comments. During the expression of its opinion, the robot exhibited weight shifts to half the numbers of participants (other robotic body movements, such as head/arm gestures were not presented). The study was between-participants design, and the measurement was conducted by questionnaires after each interaction.

### 4.1 Context Scenario

To control participants’ mental states to be frustrated in a messaging context, the following scenario and the accompanying message were given to each participant by a paper.

“You had an appointment with your ‘friend A’ to go to a movie. Today is a very hot and humid day in midsummer. You have come to meet A at the promised time, then decided to sit and wait for A in the shade under the scorching sunshine. However, A doesn’t appear and A is holding your tickets. You are already getting angry with A who does not even text you. About an hour passed after the promised meeting time, you received a message from A.”

The message reads: “I’m sorry, I am late. The appointment slipped my mind. Can you wait another hour?”

Context scenarios that make participants frustrated due to the tardiness of others is often used in psychological assessment (e.g., Rosenzweig, 1978). In designing our scenario, we modified and applied a scenario used in a Japanese study involving 183 graduate and undergraduate students (Kikuchi et al., 2008) In this scenario, the part “in the shade under the scorching sunshine” is used to control the physical load associated with each participant. To minimize both the ceiling and floor effects, a moderate level of the physical load was presented in our context scenario^2^.

In the message sent by friend A, there was a statement explaining the reason for the tardiness. To identify what reasonable statement to be included, a preliminary survey was conducted. From the survey involving 17 participants, it was revealed that recipients felt strong anger when the sender explicitly informed them of forgetting the appointment. Thus, we included the phrase in the message.

### 4.2 Design of Robot Intervention

#### 4.2.1 Verbal Expressions

The robot gave its opinion to each participant about how he/she should deal with the message. We designed three opinion types for this study based on the roles of a third party in a conflict. Third parties are known to act to support one side or not get involved in conflicts (Chaux, 2005; Hoogdalem et al., 2008).

• Opinion type 1 (suppressing anger): OMOY sided with friend A. It pacified the participant to regulate his/her anger and wait for the friend, by speaking “It is not good to get angry at the person right away, so you should wait for him/her.”

• Opinion type 2 (showing empathy): OMOY sided with the participant. It affirmed the participant’s anger and showed an empathetic attitude to the participant, by speaking “Because you have already been waiting for an hour, it is natural for you to get angry. So you don’t have to wait any longer.”

• Opinion type 3 (no comment): OMOY did not take either side of friend A nor the participant. In this case, the robot clarifies its position as a bystander by speaking “I am not involved in this topic, so I don’t have any particular opinion.”

For creating the robot’s voice, a commercial voice synthesis software called VOICEROID+ was used.

#### 4.2.2 Physical Expressions (Weight Shifts)

This study followed design recommendations by Noguchi and Tanaka (2020). They developed 36 weight-shifting patterns by using four movement parameters (target position, trajectory, speed, and repetition), and asked 18 participants about their perceptions on the emotions [based on the pleasure, arousal, and dominance (PAD) models proposed in (Mehrabian, 1996)] and intentions [based on the purposefulness dimension discussed in (Dittrich and Lea, 1994)]. The design recommendations provided useful knowledge on how each parameter should be modulated to express specific emotions and intentions. For this study, we considered the most practical weight-shifting patterns to be used from the designated scenarios.

By using the design recommendations (Noguchi and Tanaka, 2020), we designed a specific pattern to emphasize verbal expression using weight shifts. Specifically, from the 36 weight-shifting patterns tested in the study (Noguchi and Tanaka, 2020), we selected a pattern interpreted as an expression of a neutral emotion with a relatively strong intention for communicating with the user^3^. In the movement pattern, the weight was initially located at the center of the body of OMOY. Then, it moved directly and straight to the front side of the robot facing the user who holds it from the back with his/her hand. The speed parameter of the movement was designed in *fast* by following the definition in (Noguchi and Tanaka, 2020): the maximum speed at which the servo motor (SG92B) moves the weight. While the robot speaks its own opinion, as explained in Section 4.2.1, the movements were repetitively performed. The movable weight made six-round trips between the origin and the front side of its body, during which particular delays were inserted. A 100 ms pause was enacted whenever the weight reached either the origin or front of the body of the robot. In addition, a delay of 200 ms was inserted after each of the two round trips ended.

### 4.3 Participants

Ninety-four participants (51 males; 43 females; age: 18–41, *M* = 21.6, SD = 3.19) were recruited at the University of Tsukuba. They were all Japanese speakers, and every experimental material (e.g., instructions, questionnaires, and speech dialogues of the robot) were provided in Japanese. An experimental booth was set up in a cafeteria of the university, and the recruitment was conducted for passers-by. Posters and banners were placed near the booth to guide interested people in our study. Each participant got a sticky note with a print picture of OMOY on the front cover as a souvenir. The total duration required for each participant was 10–15 min. The study protocol was approved by the research ethics committee of the Faculty of Engineering, Information, and Systems of the University of Tsukuba (2019R317-2), and all participants provided informed consent.

### 4.4 Measurements

#### 4.4.1 Seriousness of the Robot

The weight shift pattern used in this study (explained in Section 4.2.2) makes the user feel strong intention of the robot. Additionally, since Ackerman et al. (2010) reported that participants using heavy clipboards rated job candidates as displaying a more serious interest in the position, it can be considered that weight shifts affect seriousness of the user. Thus, we measured the seriousness of the robot’s intention recognized by participants during the interaction, by asking “How serious was OMOY trying to tell its opinion to you?” In answering, a 9-point scale ranging from “1: It was not trying to tell it to me seriously at all” to “9: It was trying to tell it to me very seriously” was used.

#### 4.4.2 Anger

To measure the anger state of participants, we used ten question items (e.g., I am irritated, I want to shout at somebody, etc.) included in the Japanese version (Suzuki and Haruki, 1994) of a State-Anger factor in the State-Trait Anger Expression Inventory (STAXI). STAXI (Spielberger, 1988) is a well-validated psychological scale for measuring human anger based on three independent factors: State-Anger, Trait-Anger, and Anger-Expression. State-Anger represents the intensity of subjective anger at specific stimuli. The ten question items denote behavioral reactions of humans when disturbed by someone. A 4-point scale from “1: strongly disagree” to “4: strongly agree” was used to answer the questions.

To verify that the intervention by the robot influenced the user’s anger state, we measured the user’s anger state at two time points (before/after the intervention was provided)^4^. In this paper, we refer to these two anger states as pre-intervention anger (pre-anger) and post-intervention anger (post-anger), respectively. The pre-anger was measured by presenting the question items to participants and carefully instructing them to assess the mental states they held before the robot intervention (just after reading through the context scenario and hearing OMOY read out the A’s message part: “I’m sorry, I am late. The appointment slipped my mind. Can you wait another hour?”). For measuring the post-anger, we instructed them to assess the mental states they held after the robot intervention, i.e., after listening to all of OMOY’s speech contents including its opinion explained in Section 4.2.1.

#### 4.4.3 Interpersonal Motivations

We hypothesized that forgiveness provided by the participants will differ depending on the robot’s behaviors. McCullough et al. (1998) conceptualized human forgiveness from the perspective of two basic interpersonal motivations: a motivation to seek revenge or see harm come to the offender (i.e., revenge) and a motivation to avoid personal and psychological contact with the offender (i.e., avoidance). The study suggests that the reduction in revenge motivation and avoidance motivations following an interpersonal offense is closely related to forgiveness.

Thus, we measured these motivations using a Japanese-translated version of the Transgression-Related Interpersonal Motivation scale (TRIM) developed by Hashimoto and Karasawa (2012). For example, the question items include: revenge motivation – I want punishment to be given to A, I want A to get what A deserves; avoidance motivation – I would keep as much distance from A as possible, I find it difficult to act warmly toward A. In total, six question items were presented. In answering them, a 4-point scale from “1: strongly disagree” to “4: strongly agree” was used.

As earlier noted, it is important to focus on reducing those interpersonal motivations when discussing human forgiveness. Therefore, we measured these quantities twice, both before and after the intervention. These pre/post interpersonal motivations were labeled pre-intervention revenge (pre-revenge), post-intervention revenge (post-revenge), pre-intervention avoidance (pre-avoidance), and post-intervention avoidance (post-avoidance). In measuring those motivations, we provided the same instructions as were used in measuring pre/post-anger.

### 4.5 Procedure

During the recruitment of participants, we placed a public notice about our study titled “An experiment of a dialogue handheld robot.” at the cafeteria where the experimental booth was set up. If a person approached the booth and showed interest in this study, we encouraged them to take a sit on a chair placed in the booth. The study was conducted under face-to-face conditions between an experimenter and each participant. First, the participant completed a survey on their age, gender, on a form displayed on the iPad. Next, we introduced OMOY to the participant as: “Here is a handheld communication robot, named OMOY. This robot is equipped with a movable weight inside the body. One of its purpose is to mediate human messaging. Today, you will experience an example in which the robot mediates a message.”

To provide a better understanding of how the interaction with OMOY is like (e.g., about the vocal features and weight shifts), each participant was asked to listen to the self-introduction by OMOY. We instructed them to hold OMOY from the back with their non-dominant hand (similar to holding a smartphone) and to not tilt it by more than 45° to prevent it from lying down. Additionally, from pilot studies, noise from the actuators was an important consideration. Thus, participants wore headphones with a noise cancelling function (SONY WH-1000XM4) to listen to the robot. In the self-introduction, OMOY said: “Nice to meet you. Thank you for your cooperation in the test today. My name is OMOY. I will help you with your daily messaging. I read out messages received from someone and can give you my opinions when you are about to make a reply. Additionally, I can do weight shifts like this way. Best regards.” At the underlined part, OMOY performed an weight shift pattern including translational and rotational weight movements that were completely different movement patterns used in the main study.

Subsequently, the experimenter showed the context scenario paper (described in Section 4.1) to each participant while asking them to imagine their “normal friend” as friend A in the context scenario. Because it was known that people easily provide forgiveness to their intimate persons (McCullough et al., 1998), we instructed the participants that friend A is neither their best friend, boyfriend/girlfriend, nor a person with whom they want to be close. After the context scenario was given, the participants were asked to hold OMOY and wear the headphone. By sending a command to the robot through a laptop, the experimenter produced each stimulus: first, the robot auditory reported the message sent by friend A, then said its opinion with or without weight shifts. At the end of each experimental session, the experimenter passed an iPad to the participant who answered questions regarding their anger state, interpersonal motivation, and seriousness recognition.

### 4.6 Experimental Design and Statistic Analysis

Since the impressions of the robot’s intervention were expected to be different depending on the user’s affective state, we divided the participants into two groups based on high/low pre-anger using the mean value (with boundary value at 1.85). In the group of participants with low pre-anger, 52 participants were allocated, whereas 42 participants were in the group with higher pre-anger. The *t*-test ensured that the participants involved in the higher pre-anger group showed stronger pre-anger (*M* = 2.28, SD = 0.30) than the participants in the group with lower pre-anger (*M* = 1.50, SD = 0.21; *t* (70.7) = 14.3, *p* < 0.001, *d* = 0.26). To discuss the extent participants’ anger and interpersonal motivation changed before and after the robot intervention, we applied the rate of change (ROC) as dependent variables. Each value of ROC was given by the following equations:

$$ROC_{Anger}=\frac{post‐anger-pre‐anger}{pre‐anger}$$ (1)

$$ROC_{Revenge}=\frac{post‐revenge-pre‐revenge}{pre‐revenge}$$ (2)

$$ROC_{Avoid}=\frac{post‐avoid-pre‐avoid}{pre‐avoid}$$ (3)

We analyzed the effects of independent factors such as the participants’ pre-anger (high/low), opinion type, and the presence of weight shifts, on each dependent variable: seriousness recognition, post-anger, *ROC*_*Anger*_, *ROC*_*Revenge*_, and *ROC*_*Avoid*_, by performing a 2 × 3 × 2 ANOVA or ANCOVA. Suppose a significant regression coefficient of the gender factor of participants was found, we put this variable into the statistical model as covariance and performed an ANCOVA (otherwise, we used ANOVA).

## 5 Results

### 5.1 Seriousness of the Robot

We examined if participants recognized the serious intention of the robot which exhibited weight shifts. A gender difference was detected, thus we performed an ANCOVA. Results showed a significant main effect of weight shifts (*F* (1, 81) = 16.1, *p* < 0.001, ${\eta_{p}}^{2}=.17$ ) indicating that participants agreed that the robot with weight shifts was more serious (*M* = 6.10, SD = 2.58) than the case with no weight shift (*M* = 4.58, SD = 2.62). Therefore, we see that the weight shift pattern used in this study was successfully designed. Also, there was a significant main effect of the opinion type (*F* (2, 81) = 17.7, *p* < 0.001, ${\eta_{p}}^{2}=.30$ ). Then, we performed the Bonferroni test as a post hoc comparison. Results indicated that participants evaluated the robot with no comment (opinion type 3) as less serious, compared with the robots on the other two opinion types (*p* < 0.001).

### 5.2 Anger

**FIGURE 4:**
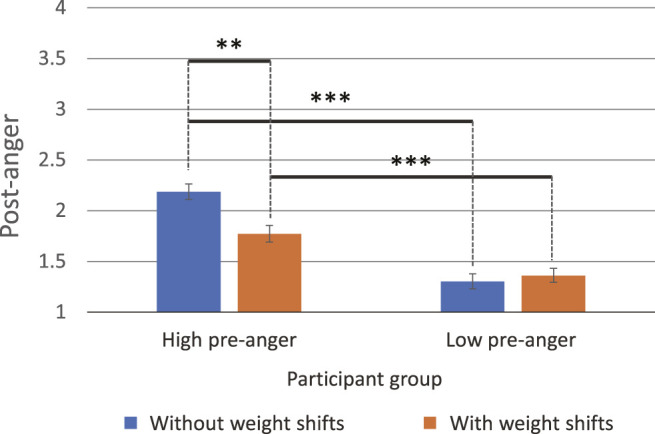
Post-anger comparison (marginal means). In the high pre-anger participant group, participants’ post-anger was significantly smaller under the condition the robot exhibited weight shifts compared with the case it exhibited no weight shift (**p < 0.01, ***p < 0.001).

First, we performed ANOVA on the post-anger variable to compare the intensity of anger that the participants held after the robot intervention. The results showed a significant interaction between the factors of the participants’ pre-anger and the presence of weight shifts (*F* (1, 82) = 9.87, *p* = 0.002, ${\eta_{p}}^{2}=.11$ ). Also, post hoc comparisons using t-tests showed a significant difference in the presence of weight shifts for participants with high pre-anger. Their post-anger was significantly smaller if the robot with weight shifts was used (*M* = 1.77, SD = 0.37), compared with the cases where the robot did not exhibit any weight shift (*M* = 2.17, SD = 0.53; *t* (40) = 2.82, *p* = 0.007, *d* = 0.46) (Figure 4). However, no significant difference was found in the participant group with low pre-anger. Also, participants having high pre-anger reported significantly larger post-anger than the cases with having low pre-anger: with weight shifts (*t* (45) = 4.29, *p* < 0.001, *d* = 0.33); without weight shifts (*t* (30.2) = 7.22, *p* < 0.001, *d* = 0.41) (Figure 4).

**FIGURE 5:**
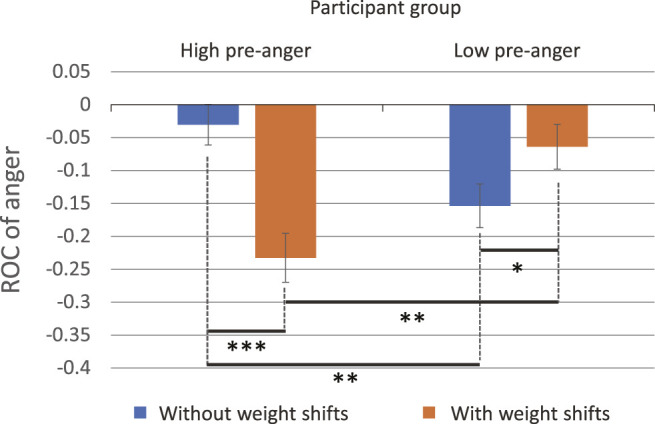
*ROC*_*Anger*_ (rate of change of anger) comparison (marginal means). The ANOVA results showed a significant interaction between the factors of pre-anger and the presence of weight shifts. Post hoc comparisons using t-tests showed significant differences in the presence of weight shifts for both groups, but the opposite effects were observed.

Next, we performed ANOVA on the dependent variable *ROC*_*Anger*_, to compare how participants’ anger level had changed over the robot intervention. The results showed a significant interaction between the factors of pre-anger and the presence of weight shifts (*F* (1, 82) = 18.5, *p* < 0.001, ${\eta_{p}}^{2}=.18$ ). Post hoc comparisons using t-tests showed significant differences in the presence of weight shifts for both groups, but the opposite effects were observed (Figure 5). In the high pre-anger group, participants’ anger was more suppressed in the condition where the robot exhibited weight shifts (*M* = − 0.23, SD = 0.18) than the case it exhibited no weight shift (*M* = − 0.035, SD = 0.15; *t* (40) = 3.86, *p* < 0.001, *d* = 0.17). However, in the low pre-anger group, participants’ anger was more suppressed in the condition where the robot exhibited no weight shift (*M* = − 0.15, SD = 0.14) than the case it exhibited weight shifts (*M* = − 0.068, SD = 0.16; *t* (50) = 2.07, *p* = 0.043, *d* = 0.15). In cases applying weight shifts, while 23% of anger was suppressed in the high pre-anger group, only 6.8% was suppressed in the low pre-anger group. The difference was statistically significant (*t* (45) = 3.28, *p* = 0.002, *d* = 0.17). In cases no weight shift is applied, 3.5% of anger was suppressed in the high pre-anger group, and 15% of anger was suppressed in the low pre-anger group. The difference was also statistically significant (*t* (45) = 2.85, *p* = 0.007, *d* = 0.14). No significant effect on the opinion type of the robot was observed.

### 5.3 Interpersonal Motivations

#### 5.3.1 Revenge Motivation

ANOVA was performed on the dependent variable *ROC*_*Revenge*_. The results revealed a significant three-way interaction (*F* (2, 82) = 3.68, *p* = 0.030, ${\eta_{p}}^{2}=.082$ ). Therefore, we performed a simple interaction test as a post-hoc comparison. An interaction was observed in both participant groups, between the presence of weight shifts and the opinion type: low pre-anger group (*F* (2, 46) = 3.06, *p* = 0.057, ${\eta_{p}}^{2}=.12$ ); high pre-anger group (*F* (2, 36) = 4.71, *p* = 0.015, ${\eta_{p}}^{2}=.21$ ).

**FIGURE 6:**
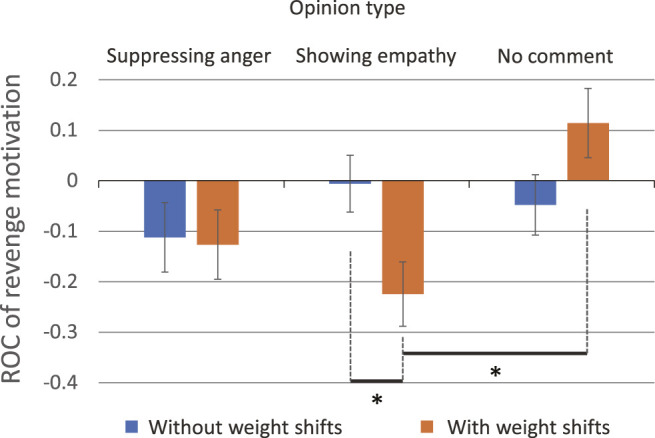
Marginal means of the rate of change (ROC) of revenge motivation in participants having high pre-anger.

In the high pre-anger group, participants’ revenge motivation was significantly reduced at opinion type 2 (showing empathy) with weight shifts (*M* = − 0.22, SD = 0.24), compared with the case without weight shifts (*M* = − 0.0057, SD = 0.16; *t* (14) = 2.21, *p* = 0.044, *d* = 0.20) (Figure 6). In addition, a significant difference was found (*F* (2, 16) = 4.49, *p* = 0.028, ${\eta_{p}}^{2}=.36$ ) between two opinion types (2 and 3) when using weight shifts (*p* = 0.029 given by Bonferroni test) (Figure 6).

**FIGURE 7:**
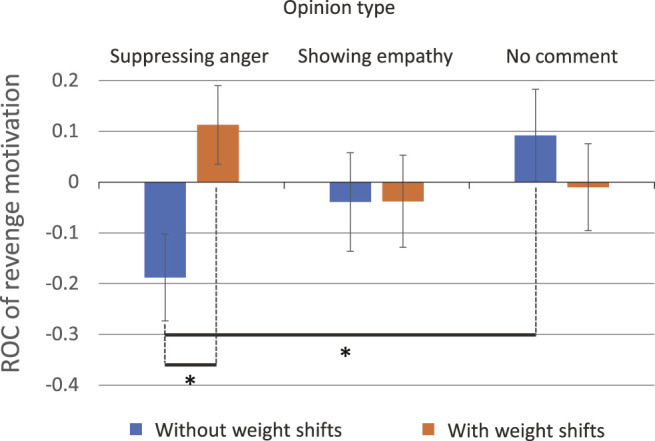
Marginal means of the rate of change (ROC) of revenge motivation in participants having low pre-anger.

In the low pre-anger group, participants’ revenge motivation was significantly reduced at opinion type 1 (suppressing anger) without weight shifts (*M* = − 0.19, SD = 0.21), compared with the case applying weight shifts (*M* = 0.11, SD = 0.33; *t* (18) = 2.36, *p* = 0.030, *d* = 0.28) (Figure 7). In addition, a significant difference was found (*F* (2, 21) = 5.07, *p* = 0.016, ${\eta_{p}}^{2}=.33$ ) between two opinion types (1 and 3) without using weight shifts (*p* = 0.014 given by Bonferroni test) (Figure 7).

#### 5.3.2 Avoidance Motivation

ANOVA was performed on the dependent variable *ROC*_*Avoid*_. The results revealed a significant interaction between participants’ pre-anger and the presence of weight shifts (*F* (1, 82) = 5.34, *p* = 0.023, ${\eta_{p}}^{2}=.061$ ). Post hoc comparisons using t-tests showed significant differences in the presence of weight shifts in both participant groups.

In the high pre-anger group, participants’ avoidance motivation was slightly suppressed when the robot exhibited weight shifts (*M* = − 0.15, SD = 0.19), compared with the case without weight shifts (*M* = − 0.058, SD = 0.15; *t* (40) = 1.68, *p* = 0.10, *d* = 0.17).

**FIGURE 8:**
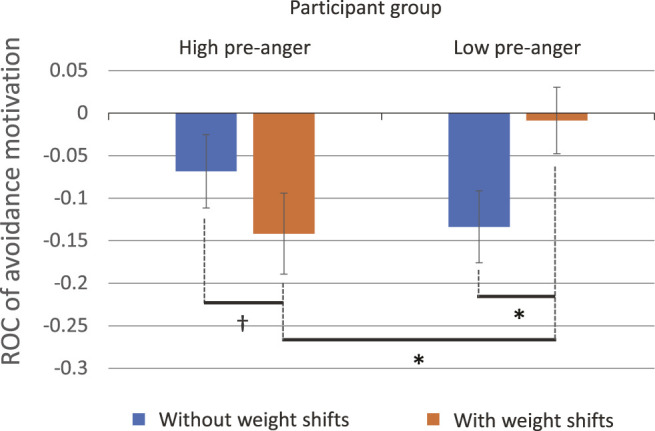
Marginal means of the rate of change (ROC) of avoidance motivation.

On the other hand, in the low pre-anger group, participants’ avoidance motivation was significantly suppressed when the robot did not exhibit weight shifts (*M* = − 0.14, SD = 0.23), compared with the case using weight shifts (*M* = − 0.0016, SD = 0.23; *t* (50) = 2.08, *p* = 0.043, *d* = 0.23) (Figure 8).

In addition, a significant difference was found between the high/low pre-anger groups when using weight shifts (*t* (45) = 3.28, *p* = 0.031, *d* = 0.22).

### 5.4 Regression Analysis

We examined whether the robot’s seriousness recognized by the participants affected their *ROC*_*Anger*_, *ROC*_*Revenge*_, and *ROC*_*Avoid*_ by the regression analysis. In the high pre-anger group, a significant regression was found for each dependent variable (*ROC*_*Anger*_ *R* ^2^ = 0.12, *F* (1, 40) = 5.20, *p* = 0.028; *ROC*_*Revenge*_ *R* ^2^ = 0.12, *F* (1, 40) = 5.21, *p* = 0.028; *ROC*_*Avoid*_ *R* ^2^ = 0.26, *F* (1, 40) = 13.8, *p* = 0.001). Regression coefficients (*ROC*_*Anger*_ *β* = − 0.028, *p* = 0.028; *ROC*_*Revenge*_ *β* = − 0.028, *p* = 0.028; *ROC*_*Avoid*_ *β* = − 0.038, *p* = 0.001) show that the seriousness recognition was negatively correlated with the participants’ anger, revenge motivation, and avoidance motivation. However, in the low pre-anger group, no significant regression was found. The results suggest that the seriousness displayed by the robot had effects only on the participants having high pre-anger.

## 6 Discussions and Limitations

Exhibiting weight shifts during text messaging was particularly effective for participants having high pre-anger. Their anger was suppressed on average by 23%. Notably, the opinion type did not make a significant difference. Because anger is a very primitive emotion and aroused instinctively, verbal messages may have been insufficient to exert a major impact to suppress it. Rather, the haptic sensations produced by the internal weight movements, or the seriousness of the robot enhanced by such movements may have been dominant in suppressing their anger.

For revenge motivation, a complex interaction was observed that involves the opinion type. In cases where the robot showed empathy to the user in words (opinion type 2) with weight shifts, the user’s revenge urge was successfully reduced by 22%. However, we found that in some cases, weight shifts promoted the motivation for revenge, thus, care must be taken in such cases. For example, when the robot expressed its unbiased position (opinion type 3) to participants having high pre-anger, their revenge motivation to the message sender was promoted by weight shifts. Overall, the opinion type 3 brought different impacts on the suppression of the user’s revenge motivation compared to the other opinion types. However, this study did not provide strong evidence for explaining why such differences were observed. Future studies are needed to gain deep understanding about the effect brought by the opinion type 3.

For avoidance motivation, the effects of weight shifts were almost consistent with those for the anger state. On average, 15% of the avoidance motivation was reduced if weight shifts were applied in participants having high pre-anger. As McCullough et al. (1998) suggested, reduction in interpersonal motivations is considered an important clue in discussing people’s forgiveness. Thus, our findings provide an evidence that the behavior of mediator robots can potentially induce forgiveness in the messaging context.

Additionally, our study results indicate that OMOY could express seriousness to the user through weight shifts. We attempt to discuss this phenomenon from the viewpoint of costly signaling theory, which has been discussed in evolutionary psychology. The theory proposes that costly signals generated by animals (including humans) may guarantee its honesty (McAndrew, 2018). For example, upon receiving a costly apology, the recipient recognizes the sincerity of the apology giver and be more likely to provide forgiveness (Ohtsubo and Watanabe, 2009). In the present study, when OMOY exhibited weight shifts, the entire body movements were visually and haptically displayed, thus the nonverbal signal was recognized as more costly than the case without weight shifts. Consequently, OMOY might be regarded as more serious when it performed weight shifts. When presenting weight shifts to the participants, OMOY had to require the force compensations from them, otherwise, it falls down. The cost for holding the robot was spent by the participants who hold it (in this case, the cost equals to the power consumed at muscles of their arms). However, even they had spent the cost, the robot was recognized as serious. This may imply that participants incorporated the cost spent by themselves for holding the robot into the estimation of the cost spent by the robot. This implication consists with the findings in the embodied perspective on cognition which suggest that the abstract concept of importance is grounded in bodily experiences of weight: heavy objects generate perceptions of importance or seriousness (Jostmann et al., 2009; Ackerman et al., 2010).

In this study, some differences were found between two participant groups (having high/low pre-anger). Generally speaking, to behave socially, we humans can intentionally suppress our anger to some extent. Neuroimaging studies revealed that our brain shows specific activation patterns when attempting to self-regulate our anger (Pietrini et al., 2000). However, the magnitude of the motivation for such self-regulation of emotions depends on the intensity of the emotion that the person has. Therefore, the participants having high pre-anger possibly had a strong motivation to suppress their anger. For those participants, the serious attitude of OMOY could have encouraged their motivation for self-regulating their anger. In contrast, the participants having low pre-anger may had little motivation for suppressing their anger, thus the serious intention of the robot exerted little influence on their psychological states. This difference may highlight a unique characteristics of weight shifts in the mechanism of reducing human anger.

A number of studies have been reporting the calming effect of touch in human, animal, and robot interaction (Eckstein et al., 2020). Touches such as stroking, holding, and pushing, so-called *social touch*, replicated by wearable devices and robots can reduce the user’s stress (Huisman et al., 2013; Kelling et al., 2016; Geva et al., 2020). As a future work, it is valuable to study if weight shifts presented by the robot have such a calming effect.

In haptic interactions between existing social robots and humans, haptic sensations occurred mostly on the user’s skin or organs close to the contact region (e.g., by temperature (Peña and Tanaka, 2020; Nie et al., 2012; Block and Kuchenbecker, 2018), texture (Hu and Hoffman, 2019), and vibration (Song and Yamada, 2017)). These sensations are cutaneous sensations activated by receptors in that region. By contrast, the robot used in this study provides a dynamic force made by a moving internal weight that activates receptors in a broader region (i.e., muscles, tendons, joints, and organs). Although this study has not provided the result of a comparison between our robot and the one with a simpler mechanism such as vibration, we have an impression that there is a clear difference between the two sensations mentioned above. However, future work is required to discuss the difference in detail with empirical evidence.

There are several limitations in our study. First, we used a simulated context scenario and the results may not hold when other scenarios are used. Second, all measurements were conducted within a relatively short time window. To supplement our findings, further empirical studies in real-world messaging are needed. Finally, the characteristics of the robot platform underline the results of this study. The results may vary under changes in parameters of the robot, such as appearance and its material. For future work, we are interested in applying the weight shift function into other handheld gadgets such as stuffed animals and cushions.

## 7 Conclusion

This study explored if a mediator robot having a weight shifting feature can affect the psychological state of the user. The robot’s body expression produced by weight shifts required no specific external components, such as arms or legs, implying that the internal weight movements potentially reduce the users’ anger, etc., without the use of rich body gestures or facial expressions. In conclusion, we suggest that weight shifting can be an effective expression modality for social mediator robots.

## Data Availability

The raw data supporting the conclusion of this article will be made available by the authors, without undue reservation.

## Footnotes

1 It gained 5 g from the original prototype reported in Noguchi and Tanaka (2020) due to the update of the weight carrier unit.

2 In case of the strongest stimulus, “stand and wait for A under the scorching sunshine,” and, in case of the weakest stimulus, “sit and wait for A in a coffee shop nearby” were used in Kikuchi et al. (2008) accordingly.

3 A video showing the weight shift pattern used in this study is available from https://youtu.be/DTkVPoI5Sc0.

4 As described in the last paragraph of Section 4.5, these were measured at once at the end of each experimental session. In measuring revenge motivation, we used three question items that had the highest factor loadings for revenge motivation in the TRIM developed by Hashimoto and Karasawa (2012).
